# A Critical Analysis of the Use of Cilgavimab plus Tixagevimab Monoclonal Antibody Cocktail (Evusheld™) for COVID-19 Prophylaxis and Treatment

**DOI:** 10.3390/v14091999

**Published:** 2022-09-09

**Authors:** Daniele Focosi, Arturo Casadevall

**Affiliations:** 1 North-Western Tuscany Blood Bank, Pisa University Hospital, 56124 Pisa, Italy; 2 Department of Medicine, Johns Hopkins School of Public Health and School of Medicine, Baltimore, MD 21218, USA

**Keywords:** cilgavimab, tixagevimab, Evusheld, LAAB, AZD7442

## Abstract

Evusheld^®^ (tixagevimab + cilgavimab; AZD7442) was the first anti-Spike monoclonal antibody (mAb) cocktail designed not only for treatment but also with pre-exposure prophylaxis in mind. The immunoglobulins were engineered for prolonged half-life by modifying the Fc fragment, thus creating a long-acting antibody (LAAB). We review here preclinical development, baseline and treatment-emergent resistance, clinical efficacy from registration trials, and real-world post-marketing evidence. The combination was initially approved for pre-exposure prophylaxis at the time of the SARS-CoV-2 Delta VOC wave based on a trial conducted in unvaccinated subjects when the Alpha VOC was dominant. Another trial also conducted at the time of the Alpha VOC wave proved efficacy as early treatment in unvaccinated patients and led to authorization at the time of the BA.4/5 VOC wave. Tixagevimab was ineffective against any Omicron sublineage, so cilgavimab has so far been the ingredient which has made a difference. Antibody monotherapy has a high risk of selecting for immune escape variants in immunocompromised patients with high viral loads, which nowadays represent the main therapeutic indication for antibody therapies. Among Omicron sublineages, cilgavimab was ineffective against BA.1, recovered efficacy against BA.2 and BA.2.12.1, but lost efficacy again against BA.4/BA.5 and BA.2.75. Our analysis indicated that Evusheld^®^ has been used during the Omicron VOC phase without robust clinical data of efficacy against this variant and suggested that several regulatory decisions regarding its use lacked consistency. There is an urgent need for new randomized controlled trials in vaccinated, immunocompromised subjects, using COVID-19 convalescent plasma as a control arm.

## 1. Introduction

AstraZeneca’s AZD7442 long-acting antibody (LAAB) cocktail (Evusheld™) consists of two monoclonal antibodies (mAbs) that bind to nonoverlapping regions of the receptor-binding domain (RBD) of the SARS-CoV-2 Spike protein. These mAbs are tixagevimab, also known as AZD8895 or COV2-2196, and cilgavimab, also known as AZD1061 or COV2-2130. Tixagevimab (PDB ID 7l7d) can be binned into the Finkelstein classification of mAbs [1] as receptor-binding motif (RBM) class III, which makes contact with nearby RBDs in addition to the one(s) they bind, and limits conformational motion of the Spike protein, with some of them locking the homotrimer in a closed state. Cilgavimab is a RBM class II mAb which is strain-specific and binds to an epitope that directly overlaps that of ACE2, but less so than RBM Class I members, such that they can bind RBD that is “up” or “down” conformation. The conformations of Spike protein and the classifications of anti-Spike mAbs have been reviewed in detail elsewhere [2].

Evusheld™ is the only combination authorized to date by both the Food and Drug Administration (FDA) and the European Medicine Agency (EMA) for pre-exposure prophylaxis of COVID-19, which is especially relevant in immunocompromised patients who fail to mount a protective immune response after multiple vaccine doses. mAb cocktails have been previously used against Ebolavirus, rabies virus (CL184, a cocktail of CR57 and CR 4098 mAbs), and SARS-CoV-2 (e.g., bamlanivimab plus etesevimab, and casirivimab plus imdevimab). Apart from preserving efficacy against viral evolution, mAbs cocktails can also retain synergistic effects by targeting nonoverlapping epitopes, but this comes at a significantly increased cost for the antibody formulation.

## 2. Preclinical Development

In designing mAb therapies for prophylaxis, a major goal is achieving high concentrations of serum immunoglobulin over extended periods of time to maintain high viral neutralizing activity. This can be done by repeated administration of antibody or by modifying the half-life of immunoglobulin molecules through protein engineering. The serum half-life of an antibody molecule is determined by the immunoglobulin Fc region, which is also responsible for complement action and interactions with Fc receptors that promote opsonophagocytosis and antibody-dependent cell cytotoxicity (ADCC). Modifying the Fc region of IgG to introduce the amino acid substitutions M252Y/S254T/T256E [YTE] increases their half-life by 2 to 4-fold, but also largely reduces ADCC [3]. This modification was used for nirsevimab (MED18897 [4,5]), which is a mAb used for the prophylaxis of respiratory syncytial virus which was applied to Evusheld™. Whether ADCC is a fundamental component of immunoglobulin antiviral activity against SARS-CoV-2 remains controversial [6,7].

Prophylactic administration of the Evusheld™ cocktail was effective in preventing infection in non-human primates and hamsters [8,9], and therapeutic administration resulted in faster clearance from the lungs. In humans, a 150 + 150 mg i.m. injection of Evusheld™ produced geometric mean titers (GMT) of neutralizing antibodies (nAb) which were >10-fold higher than in convalescents up to month 3 and 3-fold higher up to month 9. The amount of antibody measured in the nasal mucosa was only about 1 to 2% of serum level, consistent with the known low penetration of IgG into this compartment [9].

## 3. Clinical Trials

The results of the phase 1 NCT05406375 (MedImmune LLC) and NCT04896541 (AstraZeneca) in 18–55 years old Japanese individuals receiving 300–600 mg i.m. or 300–1000 mg i.v., as well as NCT05437289 in China (300 + 300 mg i.m.), have not been published yet.

The randomized controlled trials (RCT) leading to Evusheld^®^ authorizations are summarized in Table 1. Three RCTs were company-sponsored for different indications and employed i.m. administration, while a fourth was investigator-initiated and employed i.v. administration. All four RCTs were conducted at the time of the Alpha and Delta VOCs in unvaccinated subjects. None of these RCTs investigated the effect of Evusheld^®^ on viral loads during infection, albeit this parameter is not an universally accepted surrogate for efficacy [10].

In the PROVENT RCT of pre-exposure prophylaxis, Levin et al. randomized 5197 participants to receive one dose of Evusheld™ (150 + 150 mg i.m.; *n* = 3460) or placebo (*n* = 1737) from November 2020 to March 2021, a time that coincided with Alpha VOC. Symptomatic COVID-19 occurred in 0.2% (8 out of 3441) in the Evusheld™ group and in 1.0% (17 of 1731) in the placebo group (relative risk reduction, 76.7%, which evolved at 82.8% at month 6). Five cases of severe or critical COVID-19 and two COVID-19-related deaths occurred within the placebo arm [11]. Such results were communicated by AstraZeneca in a press release on 20 August 2021 [12], and the article was published in *NEJM* on 9 June 2022. The authors concluded that *“The limitations of our trial include the low number of events in smaller but important subgroups, including immunocompromised persons, so that efficacy in these groups could not be estimated”*. The drug was granted emergency use authorization (EUA) by the FDA on 8 December 2021, with the surprising indication for use in individuals having *“moderate to severely compromised immune systems”*, and a minor one being *“history of severe adverse reactions to a COVID-19 vaccine and/or component (s)”*. EMA started the rolling review on 14 October 2021, and authorized its use on 25 March 2022, while maintaining additional monitoring. The EMA indication was preexposure prophylaxis for individuals older than age 12 weighting more than 40 kg. Italy had anticipated EMA by granting authorization since 20 January 2022. Based on a presumed lack of an antibody threshold defining protection after vaccination, several European countries (e.g., AIFA in Italy on 7 June) relaxed the eligibility criteria, allowing prescription to subjects at risk for disease progression regardless of anti-Spike serostatus [13].

TACKLE was a therapeutic RCT that ran at 95 sites across Europe, USA, Latin America, and Japan during January to July 2021, a time that coincided with the Alpha VOC wave, involving 1014 non-hospitalized unvaccinated adults with WHO scores 2–3 aged 18 years or older who received Evusheld™ 300 + 300 mg i.m. or placebo within 7 days of symptom onset. Severe COVID-19 or death occurred in 4% (18 of 407 patients) in the Evusheld™ group versus 9% (37 out of 415) in the placebo group, corresponding to a relative risk reduction 50.5% [14]. AstraZeneca communicated the results in a press release on 11 October 2021 [15] and the study was published on 7 June 2022, a time when most citizens in many Western countries had received 3 vaccine doses. In Italy, AIFA expanded Evusheld™ indication to early therapy to subjects generically defined as not tolerant to small-chemical antivirals or in accordance to the epidemiological landscape [16] on the basis of these preliminary findings (including an interim analysis of the MANTICO-2 RCT (NCT05321394) aiming at testing the noninferiority of Evusheld™ 300 + 300 mg and Paxlovid^®^ versus sotrovimab in outpatients older than 50) and despite the fact that EMA had not completed a final review planned for September 2022. This decision was unfortunate, since it was announced at a time when the Evusheld^®^-resistant BA.4/5 VOC was highly dominant in Italy [17] (see below).

STORM-CHASER was a post-exposure prophylaxis RCT of 150 + 150 mg i.m., whose outcomes so far are only available via a June 2021 press-release by the vendor. A 33% (statistically not significant) relative risk reduction of symptomatic COVID-19 was found in the overall study population. The failure was mostly driven by the design both including of PCR-positive recipients at baseline (technically no longer a post-exposure prophylaxis) and counting cases occurring less than 7 days since administration. This is relevant for the i.m. route of administration, which takes longer than i.v. to achieve higher levels of serum antibody. In this regard, the time needed to generate minimum protective serum levels (2.2 µg/mL) is 6 h after 150 + 150 mg i.m. delivery (https://www.tga.gov.au/sites/default/files/evusheld-pi.pdf, accessed on 1 September 2022).

Since the results of the Evusheld arm of ACTIV-2 (NCT04518410) have not been reported yet, the only investigator-initiated RCT reported thus far is ACTIV-3 from USA. The Therapeutics for Inpatients with COVID-19 (TICO) Study Group Investigated Evusheld™ as treatment for patients hospitalized with COVID-19 from 10 February 2021, to 30 September 2021, enrolling 1455 adults with symptoms for a median of 8 days, of whom 47% were seronegative, 15% were vaccinated with 2 doses, and 12% were vaccinated with one dose. These patients were hospitalized with COVID-19 WHO stage 4–5 at 81 sites across Europe, USA, Uganda, and Singapore and were randomized 1:1 ratio to standard of careplus intravenous tixagevimab 300 mg–cilgavimab 300 mg (*n* = 710) or standard of care plus placebo (*n* = 707). There were no differences in sustained recovery at 3 months, regardless of serostatus, but Evusheld™ led to lower mortality (9% vs. 12%) [18].

The above recounting of the information available for Evusheld™ shows the absence of clinical efficacy information during the omicron VOC phase of the pandemic. Consequently, there is an absolute need for confirmatory RCTs in vaccinated adults at the time of Omicron waves, but there are limitations on what can be done. In this regard, it is reasonable to avoid placebo-based control arms at this stage, which limits options largely to observational studies, but we are concerned about the absence of studies evaluating this drug against alternative treatments. In this regard, Astra-Zeneca-sponsored NCT05184062 RCT in China could have investigated Evusheld™ 300 + 300 mg i.v. versus placebo but is currently not recruiting. Similarly, the investigator-initiated DisCoVeRy RCT in France (NCT04315948, part of the WHO Solidarity Trial) could have investigated Evusheld™ versus other treatments or placebo but is also currently not recruiting.

Most second-generation trials initiated so far are observational in nature, the largest being COVIMAB in France (NCT05439044) and EVOLVE in UAE (NCT05315323). Most of them will focus on pre-exposure prophylaxis in immunocompromised patients, with some including any cause of immunosuppression (e.g., PREP in USA (NCT05461378) and PRECOVIM in France (NCT05216588) and others being focused on specific patient subgroups (e.g., NCT05438498 in cancer patients in USA or TIXCI-TRANS in solid organ transplant recipients (SOTR) in France (NCT05234398)).

More trials are ongoing in pediatric patients. The phase I NCT05281601 will investigate safety of i.m. or i.v. Evusheld™ in patients aged ≥29 weeks gestational age to <18 years, while the phase 2 NCT05375760 (ENDURE) will investigate pre-exposure prophylaxis in moderately-to-severely immunocompromised patients aged >12 years with Evusheld™ 300 + 300 mg i.m. every 3 versus 6 months.

There is also a reasonable attempt to investigate the two ingredients individually, given the likelihood of resistance emerging. Astra-Zeneca-sponsored NCT05166421 RCT is investigating individual ingredients versus the cocktail as preexposure prophylaxis in adults >18 years.

The safety profile has been very good so far, with only a few reports of hypersensitivity reactions [19] and myalgia [20].

## 4. Baseline and Treatment-Emergent In Vitro Inefficacy against Omicron Sublineages

**Figure 1:**
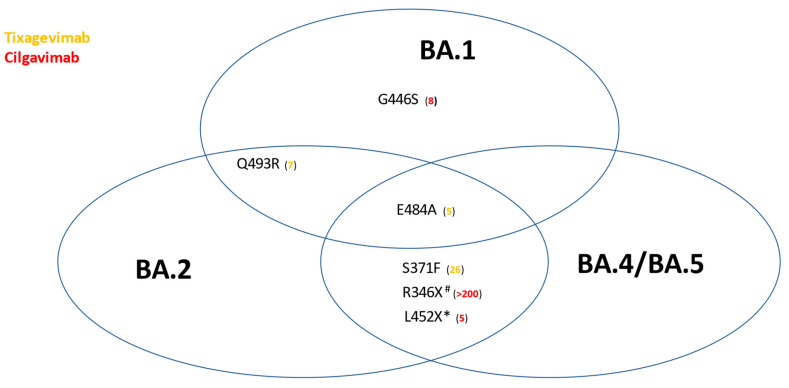
Impact of selected Spike protein mutations on the in vitro efficacy of Evusheld™. Numbers in parentheses represent fold-reductions in the geometric mean titer of neutralizing antibody titers. Only mutations for which the majority of studies are concordant are reported. Sourced from https://covdb.stanford.edu/page/susceptibility-data/ (accessed on 2 September 2022). * L452R is universal among BA.4/5, but occurs only in BA.2.11, BA.2.35, BA.2.75.4, BA.2.77, while L452Q occurs only in BA.2.12.1. # R346I in BA.5.9, R346S in BA.4.7 and BF.13, R346T in BA.1.23, BA.2.9.4, BA.2.80, BA.2.82, BA.4.1.8, BA.4.6, BF.7 and BF.11.

The Stanford University Coronavirus Antiviral and Resistance Database (https://covdb.stanford.edu/search-drdb, accessed on 1 September 2022) shows that tixagevimab has no efficacy against S371F, F486S/V, Q493R, and Q498R, while cilgavimab has no efficacy against R346X, E406W [21], K444E/Q/R, and V445A. In addition, none of the 2 mAbs have efficacy against E484A/K (Figure 1). Resistance can be determined by either viral neutralization assays [22] or biolayer interferometry [23]. When it comes to Omicron sublineages, these mutations compromise the efficacy of the ingredients (Table 2 and Table S1).

Against BA.1, cilgavimab was ineffective (median 58-fold reduction in GMT compared to WA.1 in authentic live SARS-CoV-2 neutralization tests (VNTs), 570-fold reduction in pseudoVNT), as well as tixagevimab (230-fold reduction in authentic VNT, 1642-fold reduction in pseudoVNT), and the cocktail was 274-fold resistant in authentic VNT [24,25] and 302-fold resistant in pseudoVNT.

Against BA.2, cilgavimab recovered full efficacy (median 2-fold reduction in both authentic VNT and pseudoVNT [26]). Despite tixagevimab remaining ineffective (median 1000-fold reduction in pseudoVNT), the cocktail recovered some efficacy (5-fold reduction in authentic VNT and 8-fold reduction in pseudoVNT). Against second-generation BA.2 sublineages the situation varied: in pseudoVNT, cilgavimab was just 3-fold less effective against BA.2.12.1, but it was 34-fold less effective against BA.2.75: combined with the persisting absolute inefficacy of tixagevimab (382- and 1000-fold less effective against BA.2.12.1 and BA.2.75, respectively), the cocktail was left with partial efficacy (10-fold reduction against BA.2.12.1, and 54-fold reduction against BA.2.75).

Against BA.4/5 cilgavimab showed a 9-fold reduction, while tixagevimab was again ineffective (1000-fold reduction in efficacy), leaving the cocktail with a 21-fold reduced efficacy. The efficacy is particularly compromised against emerging R346X-carrying BA.4/5 sublineages (IC_50_ > 10,000) that are dominant as of Summer 2022 (BA.4.6, BA.4.7, and BA.5.9). Deep mutations scanning (DMS) studies have also been conducted [27,28].

Irrespective of Omicron lineage, the antiviral activity no longer comes from a cocktail but rather from a single mAb, namely cilgavimab. This is important because the experience with other mAb monotherapies has shown that immune escape is likely to be expected [2].

Despite the in vitro reductions in activity, there is hope that some clinical efficacy remains. Stadler et al. showed that for many of the anti-Spike mAb regimens, clinically administered doses are between 7 and >1000 fold higher than necessary to neutralize 90% wild-type SARS-CoV-2, suggesting potentially preserved efficacy against Omicron sublineages despite reduced in vitro neutralization [29]. Accordingly, Evusheld™ reduced BA.1, BA.1.1, and BA.2 lung infection in human ACE2-transgenic K18 miceI when administered for prophylaxis of therapy [30], but these encouraging results have not been confirmed in clinical experience (see paragraph below).

Treatment-emergent immune escape studies in vitro show that cilgavimab selects for N74K [27], R346I [27,31], K444Q/E/R [31,32], and S686G [27], while tixagevimab selects for G476D and N487D [32]. The cocktail selects for R346G, E484K, and F486V [32]. No in vivo study has been reported yet.

## 5. Post-Marketing Clinical Studies

Data on efficacy and safety in vaccinated subjects during the Omicron waves are thus far limited to a few retrospective cohort studies, focusing exclusively on preexposure prophylaxis in immunocompromised recipients.

The first concern that emerged was about pharmacokinetics. Only 9.5% of 63 kidney transplant recipients (KTR) who received prophylactic Evusheld™ (150 + 150 mg i.m.) were able to neutralize BA.1 at 1 month, compared to 71% of convalescents and 2.6% of those who received Ronapreve™. Convalescents displayed higher nAb levels than those who received Evusheld™. The high interindividual variability in anti-RBD IgG titers seen after Evusheld™ could be accounted for by the recipient body mass index [33].

The second concern was about efficacy. Stuver et al. at MSKCC reported breakthrough infections in 2 out of 52 oncohematological patients who had received Evusheld™ (150 + 150 mg i.m.) (3.8%) [34]. In Israel, Kertes et al. reported that 29 out of 825 immunocompromised patients (3.5%) became infected with BA.1 compared to 308 (7.2%) of 4299 non-randomized immunocompromised patients not administered Evusheld™ (OR 0.51): 1 person in the Evusheld™ group (0.1%) was hospitalized for COVID-19 compared to 27 (0.6%) in the non-administered group (*p* = 0.07) [34]. In a large cohort study, Nguyen et al. in France reported breakthrough infection in 49 out of 1112 (4.4%) immunocompromised patients at least 5 days following treatment with Evusheld™. Benotmane et al., in France, showed that among the 416 KTRs who received prophylactic Evusheld™ (150 + 150 mg i.m.) at the time of BA.1, 39 (9.4%) developed symptomatic COVID-19, requiring hospitalization and ICU admission in 36% and 3 patients, respectively [35].

These findings suggested that Evusheld™ 150 + 150 mg was not sufficient against BA.1 and BA.1.1, which led the FDA to recommend doubling the recommended dose (300 + 300 mg) from 24 February 2022 [36]. On 22 March 2022, in a Dear Healthcare Provider letter, AstraZeneca recommended that all individuals who received only the previously authorized initial dose (150 + 150 mg) should immediately receive an additional Evusheld™ dose. If the patient had received their initial dose >3 months ago, the patient should then receive an additional dose of 300 + 300 mg [37]. A similar recommendation has not been made by EMA at the time of writing. Since 29 June 2022, the FDA has allowed repeated courses of Evusheld™ 300 + 300 mg every 6 months. The approved dose for treatment remains 300 + 300 mg in both the FDA and EMA authorizations.

Cohort clinical studies conducted at the time of the BA.2 VOC revealed that Evusheld™ performed slightly better, in line with in vitro evidence showing restored activity of cilgavimab against BA.2. Bruet et al. in France reported 9-fold reduced efficacy compared to Delta and recorded 4 breakthrough Omicron infections among 29 immunocompromised individuals [26]. Karaba et al., in Baltimore, showed that among 61 SOTR receiving tixagevimab plus cilgavimab 300 + 300 mg, BA.1 neutralization was low and did not significantly increase after Evusheld™ administration. In contrast, BA.2 neutralization increased from 7% to 72% of participants post-Evusheld™ (*p* < 0.001). Evusheld™ increased anti-RBD levels, but BA.1 neutralizing activity was minimal [38]. Al Jurdi et al., in Boston, conducted a retrospective cohort study comparing 222 SOTR who received Evusheld™ for pre-exposure prophylaxis in the first 3 months of 2022 and 222 age-matched vaccinated SOTR controls and reported that the 60-day incidence of breakthrough infection was 1.8% in the Evusheld™ group and 4.7% in the control group (*p* = 0.045) [39]. At follow-up, breakthrough infections occurred in 11 (5%) within the Evusheld™ arm and in 32 (14%) within the control group (*p* < 0.001). In the Evusheld™ group, SOTRs who received the 150 + 150 mg dose had a higher incidence of breakthrough infections compared to those who received the 300 + 300 mg dose (*p* = 0.025) [39]. Young-Xu et al. found that, compared to 251,756 propensity-matched immunocompromised or at-risk historical controls at Veteran Affairs Healthcare Systems, 1848 Evusheld™-treated patients had a lower incidence of SARS-CoV-2 infection (HR 0.34), COVID-19 hospitalization (HR 0.13), and all-cause mortality (HR 0.36) [40].

There are not clinical studies reported so far against the currently dominant BA.4/BA.5 lineages (and in particular against the emerging sublineages BA.4.6, BA.4.7, and BA.5.9) and second-generation BA.2 sublineages (e.g., BA.2.12.1, BA.2.75), which again in vitro are poorly neutralized by Evusheld™ because of the R346X and L452X mutations [41]. Bruel et al. analyzed 121 sera from 40 immunocompromised individuals up to 6 months after infusion of 300 or 600 mg of Evusheld™. Antibody responses against BA.5 decayed slightly faster than against BA.2 in 8 patients. Such a decrease may be negligible when the Evusheld™ antibodies are measured alone but is more visible in the serum [3].

On the basis of this evidence, the UK government was advised by Rapid C-19 not to procure Evusheld™, contrary to Italy, Canada, France, and Israel [42].

## 6. Conclusions

The development and clinical deployment of the Evusheld™ mAb cocktail during the COVID-19 pandemic was a successful application of basic scientific knowledge in antibody engineering and viral neutralization that undoubtedly saved many lives. However, we are struck by gaping holes in clinical efficacy data and inconsistent regulatory decisions. We note that all clinical efficacy data from RCTs comes from cohorts that were not vaccinated, minimally immunocompromised, and were studied when the circulating virus was susceptible to neutralization by both mAbs. Since then, no RCT has been launched to replicate efficacy in vaccinated, immunocompromised patients, who represents the vast majority of Evusheld™ prescriptions these days. We do not know if the presence of vaccine-elicited antibodies to SARS-CoV-2 affects the pharmacokinetics of the Evusheld™ cocktail. Despite this, regulatory authorities decided to authorize its use. Unfortunately, this happened at the time Omicron VOC emerged, which defeated one of the two mAbs in the cocktail and immediately led to the recommendation of doubling the dose, a decision based exclusively on in vitro assays. Despite such corrective action, the available in vitro and in vivo evidence suggest that Evusheld™ risks less than desirable clinical efficacy against the currently dominant Omicron sublineages BA.4/5. This is even more relevant in immunocompromised subjects, who start with very high viral loads. We recognize that regulatory agencies acted in the rapidly evolving pandemic environment and were more willing to “proceed on risk” under such circumstances that it would be acceptable in a normal situation, but reassessment is urgently needed to avoid ongoing malpractices in mAb prescriptions [17,43].

We note that alternative poyclonal antibody preparations in the form of convalescent plasma from vaccinated individuals, such as VaxPlasma, are available that have high neutralizing activity against all Omicron variants [44]. Given that there is only one active mAb in the Evusheld™ cocktail against the earlier Omicron variants and that monotherapy can select for resistance in immunocompromised patients, polyclonal preparations such as VaxPlasma have inherent advantages in this population and should be currently favored over Evusheld™ for these patients [45]. In the meanwhile, trials of extended schedules of small-molecule antivirals will assess safety and efficacy (e.g., NCT05438602).

**Table 1:** Synopsis of randomized controlled trials using Evusheld™.

NCT (Ref)	Indication	Treatment Arm (*n*)	Control Arm (*n*)	Main Efficacy Outcome(s)
NCT04625725 (PROVENT) [11]	pre-exposure prophylaxis	3433	placebo (1717)	Symptomatic COVID-19 in 8 out of 3441 (0.2%) in the Evusheld™ group and in 17 out of 1731 (1.0%) in the placebo group (relative risk reduction, 76.7%); extended follow-up at a median of 6 months showed a relative risk reduction of 82.8%). 5 cases of severe or critical COVID-19 and 2 COVID-19-related deaths occurred, all in the placebo group.
NCT04625972 (STORM CHASER) [46]	post-exposure prophylaxis	749	placebo (372)	33% (statistically not significant) relative risk reduction of symptomatic COVID-19 in the overall population 73% relative risk reduction for symptomatic COVID-19 in those PCR-negative at baseline (92% reduction for cases >7 days following dosing; 51% reduction for cases up to 7 days following dosing)
NCT04723394 (TACKLE) [14]	outpatient therapy	452	placebo (451)	Progression of COVID-19 or death at day 29 was 4% in the treatment group (3.5% if administered within day 5) vs. 9% in the placebo group (relative risk reduction of 50.5%)
NCT04501978 (ACTIV-3)	inpatient therapy	710	placebo (707)	Sustained recovery was 89% for Evusheld™ and 86% for placebo at day 90 regardless of serostatus. Mortality was 9% (61) with Evusheld™ versus 12% (86) with placebo

**Table 2:** In vitro efficacy of tixagevimab and cilgavimab against SARS-CoV-2 variants of concern (VOC). Arrows indicate fold-reductions in neutralizing activity compared to wild-type D614G strain (e.g., Wuhan-Hu-1, USA-WA1/2020, B.1, or other reference strains) (=no reduction; ↓: 1–3 fold reduction; ↓↓: 3–5 fold reduction; ↓↓↓: >5 fold reduction). PHE: Public Health England.

WHO VOC	Alpha	Beta	Gamma	Delta	Omicron							
PANGOLIN name	B.1.1.7	B.1.351	P.1	B.1.617.2	BA.1	BA.2	BA.2.12.1	BA.2.75	BA.4/BA.5	BA.4.6	BA.4.7	BA.5.9
NextStrain name	20I/S:501Y.V1	20H/S:501Y.V2	20J/S:501Y.V3	21A/S:478K and descendants 21I/21J	21K (descendant of 21M)	21L (descendant of 21M)	22C	22D	22A/22B	-	-	-
UKHSA/PHE name	VOC-20DEC-01	VOC-20DEC-02	VOC-21JAN-02	VUI-21APR02	VUI-21NOV-01	VUI-22JAN-01	-	V-22JUL-1	VOC-22APR-03/VOC-22APR-04	-	-	-
**GISAID name**	GRY (formerly GR/501Y.V1)	GH/501Y.V2	GR/501Y.V3	G/452R.V3	GRA (formerly GR/484A)							
tixagevimab /AZD8895/COV2-2196	↓↓↓ [47]	↓↓↓ [47]	= [48]	= [27,49,50,51,52,53,54]	↓↓↓ [25,41,53,54,55,56,57,58,59,60]	↓↓↓ [26,41,58,59,60,61,62,63,64] (including BA.2.11, BA.2 L452Q, BA.2 S704L, BA.2 HV69-70del, BA.2 F486V, BA.2 R493Q [60])	↓↓↓ [41,58,60,63,64]	↓↓↓ [62,63,64,65,66]	↓↓↓ [41,58,59,60,62,63,64]	↓↓↓ [67,68]	↓↓↓ [67,68]	↓↓↓ [67,68]
cilgavimab /AZD1061/COV2-2130	= [47]	= [47]	= [48]	↓ [27,49,50,51,52,53,54]	↓↓↓ [24,25,53,55,56,57,58,59,60]	↓ [26,41,58,59,60,61,62,63,64] (including BA.2.11, BA.2 L452Q, BA.2 S704L, BA.2 HV69-70del, BA.2 F486V, BA.2 R493Q [60])	↓ [41,58,60,63,64]	↓↓↓ [62,63,64,65,66]	↓↓ [41,58,59,60,62,63,64,69]	↓↓↓ [67,68]	↓↓↓ [67,68]	↓↓↓ [67,68]

## Data Availability

No new data were created or analyzed in this study. Data sharing is not applicable to this article.

## Supplementary Materials

The following supporting information can be downloaded at: https://www.mdpi.com/article/10.3390/v14091999/s1, Table S1: Summary of IC50 geomeans and median fold reductions for either tixagevimab or cilvagimab against Omicron sublineages.

## Abbreviations

EUA	emergence use authorization
RBD	receptor binding domain
VOC	variant of concern
RCT	randomized controlled trial
